# From leisure motivation to recreation specialization: a study on the psychological mechanism of sustainable participation of ice and snow sports participants

**DOI:** 10.3389/fpsyg.2025.1484550

**Published:** 2025-11-20

**Authors:** Yuan Liang, Jinglye Li, Zhaohong Wang

**Affiliations:** 1 Beijing Normal University, College of P.E. and Sports, Beijing, China; 2 Beijing Dance Academy, Beijing, China; 3 Department of P.E., Renming University of China, Beijing, China

**Keywords:** ice and snow leisure, leisure motivation, leisure constraints, leisure negotiation, recreation specialization

## Abstract

**Introduction:**

This study explores ice and snow leisure behavior, a key topic in China’s ice and snow industry development. Using 914 Chinese participants in leisure-related ice and snow activities as samples, it aims to identify the mechanisms of such behavior and validate hypotheses in a theoretical model.

**Methods:**

Structural equation modeling (SEM) was used to analyze the “Leisure Motivation—Leisure Constraints—Leisure Negotiation—Recreation Specialization” model, verifying nine hypotheses on direct and mediating effects.

**Results:**

(1) Leisure motivation is the main internal driver for ice and snow sports participation; (2) Leisure negotiation strategies are critical to promoting long-term mass participation by alleviating constraints; (3) Restrictive factors (e.g., accessibility, cost) significantly hinder the ice and snow industry’s sustainable development.

**Discussion/Conclusions:**

Based on empirical findings, a multidimensional model deconstructing leisure motivation is proposed to enhance internal (e.g., motivation cultivation) and external (e.g., policy) support. Targeted suggestions are offered to boost leisure flexibility and mitigate constraints, providing references for China’s ice and snow leisure industry.

## 1 Introduction

The successful hosting of the Beijing Winter Olympics fulfilled the solemn promise of “300 million people participating in ice and snow” sport. The concept of ice and snow has penetrated into the homes of ordinary people in China, and the spirit of ice and snow in the Winter Olympics has made ice and snow sports a fashion project in which the public can participate. However, the primary “experiential” and “follow-up” methods of participating in ice and snow sports are still dominant, and the public has not yet established a habitual behavior order based on embodied experience (Wang, 2022). Broadening the group of participants and cultivating stable recreation professionals are necessary paths for the sustainable development of the ice and snow industry. Driven by interest and motivation, the key aim of this study is to deeply analyze the influence path of mass participation behavior and leisure specialization. Leisure theory posits that the multiple motivations of leisure activities are the antecedents that influence the behavioral mechanism and that the constraints of subject-object causes hinder the progress of leisure activities (Nye, 1990); however, by implementing alternative strategies, these restrictions can be assessed, adapted, mitigated, or eliminated to promote ultimate participation (Jackson et al., 1993). Recreation specialization is a deep advancement in terms of participation and is a sign of the mature development of leisure. Compared with foreign research on the mechanism underlying the influence of ice and snow recreation specialization, domestic research is not sufficient. Accordingly, this study takes people participating in leisure-related ice and snow projects in Beijing as a research sample, explores their leisure behavior, constructs a leisure behavior scale, and verifies the “leisure motivation→ leisure constraints→ leisure flexibility→ recreation specialization” theoretical model by using scientific empirical methods to analyze the individual motivation of such people, subjective and objective interventions and alternative content to provide theoretical support for the sustainable development of ice and snow for the public.

## 2 Literature review

### 2.1 Leisure theory

Leisure is defined as a freely chosen activity that involves meaningful, enjoyable experiences (Godbey, 1981) and a process of enjoyment and self-expression. Due to the great satisfaction of material life, leisure has become an important element in the modern lifestyle. Caldwell (2005a) divided leisure into active leisure (which requires a certain degree of physical exertion), passive leisure (including rest, recovery and quiet activities) and social leisure (which focuses on social interaction), which allows people to experience a feeling of concentration and comfort. Leisure is inherently imperceptible and not a necessity; however, several studies have found that leisure activities have positive effects on individual subjective perceptions (Schmiedeberg and Schroeder, 2017). Ryan and Deci (2000) agreed that leisure helps promote autonomy, social relationships, and optimism, thereby enhancing stress coping resources and personal well-being in the context of self-determination theory. Therefore, this study takes ice and snow leisure projects as its research object to explore the endogenous theory of leisure, including leisure motivation, leisure constraints and the corresponding adaptation strategies, and leisure specialization, with the goal of deeply understanding the mechanism and internal logic underlying the behavior of people in the context of leisure-related ice and snow activities.

### 2.2 Leisure motivation

Motivation is considered to be the intrinsic force that drives individual behavior and can help explain observed changes in individual behavior (Petri and Govern, 2003). In 1980, Crandall (1980) introduced the concept of leisure motivation, which is defined as a need, reason or satisfaction to encourage participation in leisure activities. Leisure motivation provides participants with opportunities for self-determined behavior, interest exploration, identity development, skill development, and the pursuit of meaningful and personal expression experiences (Caldwell, 2005b), and it has become an important aspect of modern leisure research. Based on in-depth explorations of external nature and the human body itself, leisure scholars have investigated the profound connotations of leisure motivation from a multidisciplinary perspective rooted in psychology, anthropology, and sociology. The theory of happiness is based on consideration of the intrinsic motivation of leisure and the satisfaction that follows leisure activities, and participants are motivated by the quality of their experience when participating in leisure activities, thus leading to continuous participation behavior (Guha, 2009). Self-determination theory focuses on the internal psychological needs of individuals and claims that the choice of leisure mode is a dynamic and progressive process that involves the individual learning and exploring various leisure modes, experiencing the satisfaction generated by self-determination and internal regulation, and then continuing to participate (Deci and Ryan, 2000; Ryan, 1993). Dann (1977) claimed that leisure motivation is affected by a combination of internal forces (push) and external forces (pull) and constitutes the intrinsic motivation of individuals to participate in leisure methods; in this context, the pull is derived from the destination attribute and the quality attraction of leisure activities. Wang et al. (2020) empirically investigated the factors that influence the push (i.e., social motivation) and pull (i.e., ski resort attribute preferences) factors that affect the level of recreational skiing participation in China with the goal of providing an effective basis for increasing the gravity and improving the service quality of ski resorts.

The theory of leisure motivation has laid the foundation for more diverse leisure styles and a wide range of groups, and the content of this research has been extended to include many fields; corresponding research methods have gradually become more diverse. Özdemir (2020) elucidated the intrinsic leisure motivation of college students, an important group of leisure participants, and identified the relationships and differences between some variables and intrinsic leisure motivation. Dillard (2011) found that the four core motivations for leisure are escapism, strengthening relationships, improving self-efficacy, and obtaining a sense of victory. Tretyakevich and Maggi (2012) evaluated the factors motivating businesspersons to participate in various types of leisure and concluded that external support such as environmental space, cultural diversity, the novelty of leisure, and personal relaxation are all motivational factors that encourage businesspersons to relax. Snepenger (2006) evaluated six competing leisure motivations using confirmatory factor analysis and found that leisure participants exhibited high motivation with regard to self-challenge and escapism. By studying the leisure lifestyle of Hangzhou residents, Chen et al. (2018) found that residents’ leisure behavior and leisure motivation are related.

Exploring leisure motivations and the corresponding influencing factors can facilitate the development of deeper leisure behaviors in leisure activity settings and provide valuable information for the managers of leisure activity projects (Kim et al., 2019). Accordingly, this study introduces the theory of leisure motivation to explore the behavioral will of people who engage in ice and snow leisure activities by reference to rigorous empirical evidence.

### 2.3 Leisure restrictions

The popularity of leisure activities has greatly enhanced the vitality of modern lifestyles, but the development process has not been smooth. Duane and Crawford (1987) considered the factors that promote and hinder the development of leisure activities, such as lack of interest in leisure, wealth constraints, and time constraints; based on this consideration, they abstracted the hierarchical model of leisure constraints, arguing that leisure constraints include any factors that intervene in the relationship between the preference for a certain activity and participation in that activity, including the three elements of personal internal constraints, interpersonal constraints and structural constraints (Godbey et al., 2010). They subsequently expanded and extended this model with regard to the order of ranking (Crawford et al., 1991). Leisure constraints have been widely used to study leisure constraints in various industries. Leslie et al. (1994) studied the relationships among leisure style, self-esteem, gender and social status among adolescents and verified the three-dimensional hierarchical model using confirmatory factor analysis; the results of this analysis provided supporting evidence for the model scale. However, Hawkins et al. (1999) in their investigation of leisure activities in the context of mentally lagging adults, failed to confirm the hypothetical hierarchy among the three constraint categories, suggesting that individual constraints were more prominent than interpersonal and structural factors, a result also reported by Jackson and Henderson (1995), additional support for this point has also been found in research on women’s leisure. Scott and Munson (1994) also highlighted possible interactions within individuals, relationships, and structural constraints. McCarville and Smale (1993) investigated the perceived limits of recreational participation in five activity domains (including physical activity and exercise, arts and entertainment, hobbies, social activities, and family recreation) and found that constraints tended to be unevenly distributed across groups in total. These heterogeneous studies have fully indicated that leisure constraints exhibit a great deal of diversity, a finding which is of great value for attempts to overcome obstacles to leisure participation, enrich leisure experience, develop precise policies, and expand the scope of participants in leisure. Therefore, this paper adopts the hierarchical model of leisure constraints as a theoretical basis and introduces research on ice and snow leisure methods to explore in depth the internal constraints associated with ice and snow recreation and provide a scientific basis for the managers of services that facilitate ice and snow activities.

### 2.4 Leisure negotiation

**Figure 1:**
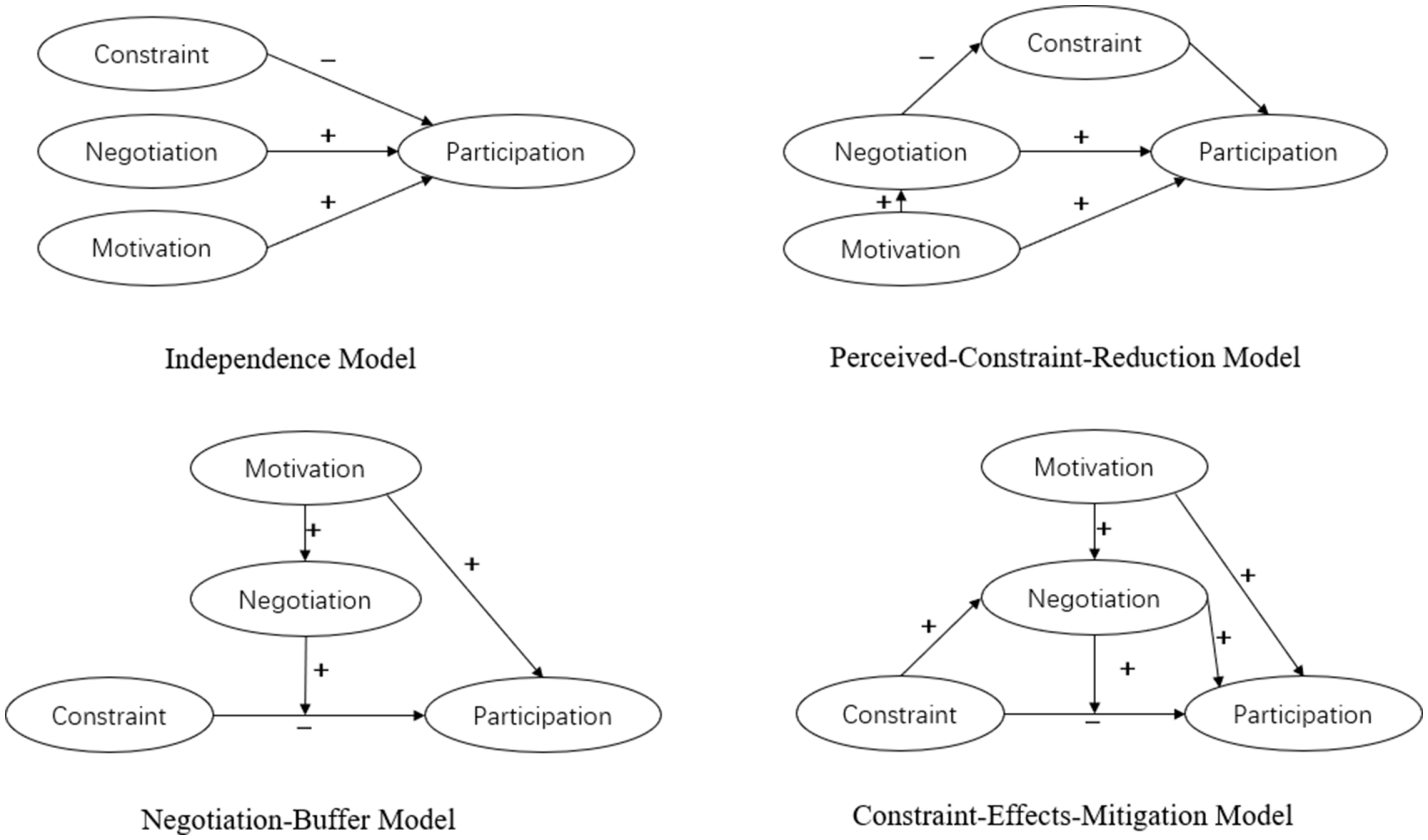
Model of leisure theory.

Leisure constraints are believed to inhibit participation in activities or limit satisfaction, and obstacles inevitably lead to nonparticipation. However, as in-depth research on leisure has shown that constraints are not insurmountable, Jackson et al. (1993) synthesized existing research, suggested a series of propositions to outline the negotiation process of constraints and proposed the core idea that leisure participation does not depend on the absence of constraints but on the implementation of flexibility through negotiation, which is evident in the fact that when people recognize the fact that they have encountered constraints, certain strategies to overcome them may be proposed to assess, adapt, mitigate or eliminate those constraints. For example, Scott (1991) found that bridge players overcome time constraints, manage schedules, and develop new operational skills when given the opportunity to participate. Kay and Jackson (1991) found that when people engaging in leisure activities face financial constraints, they look for more affordable ways to participate; when faced with time constraints, the time and work they spend on housework are reduced. With regard to constraints, Jackson and Rucks (1995) pioneered the idea of generalizing alternative strategies into cognitive and behavioral strategies, specifically identifying time management, skill acquisition, changing relationships, improving finances, and changing leisure desires. Hubbard and Mannell (2001) extended the scope of leisure theory and tested four competing models using validation factor analysis and structural equation models, including independent models, workaround-buffer models, limit-effects buffer models and perception-constraint-reduction models, each of which contains a structure consisting of leisure motivation, leisure constraints, adaptation, and leisure participation, as shown in Figure 1.

Among the model results, the constraint-effects mitigation model fits the data best, indicating that encountering leisure constraints motivate people to adopt workaround strategies, thereby mitigating the negative effects of leisure constraints. This fact also explains the important mediating role that workarounds play in the relationship between motivation and engagement. Based on the competitive model, Dave explicated the course of outdoor entertainment based on the leisure constraints variable, further confirming that the constraint variable process is a dynamic interaction that promotes the influence of participation in outdoor entertainment (White, 2008). Kono et al. (2021), who obtained online survey data from 618 Japanese, European, and Canadian leisure practitioners, claimed that a variety of alternative strategies are needed to address different types of constraints. As an outdoor leisure sport, skiing has certain prerequisites for participation, such as weather, price, distance, skills, and sports culture, which may be factors that restrict participation (Zhang, 2022). Therefore, this paper adopts the theoretical thinking of restriction adaptation, explores the behavior of people in the context of ice and snow leisure activities in China in depth, and tries to explore alternative strategies to limit the participation of skiers.

#### 2.4.1 Recreation specialization

Due to the development of the social economy, leisure activities are gradually becoming integrated into the lifestyle of the public, and participation in sailing, riding, hunting, skiing, fishing and other recreational projects is increasing rapidly. Unlike traditional primary games, some committed enthusiasts use complex ways to deepen their connection with these activities, thus entering the stage of professional development (Liu and Lou, 2019). In response to this phenomenon, Bryan (1977) developed the concept of recreational specialization in 1977 as a way to identify, describe, and plan these recreation groups, defining specialization as a continuum of behaviors, ranging from general to specific, that reflect the equipment, skills, experiences, and value orientations involved in motor and activity setting preferences. Sports sociologists began to use the term specialization at an early point to describe the tendency of athletes to participate heavily in a single sport to the exclusion of others and later applied it to a variety of leisure modalities.

As a basic element of behavior (Hill and Simons, 1989), people who engage in recreation experience a dynamic process of leisure socialization, and their mode of movement or participating in such hobbies depends on their stage of development in the activity in question (Virden and Schreyer, 1988); that is, their level of recreation specialization increases with the improvement of their skills, equipment, emotions and other elements. Therefore, some scholars have claimed that the process of recreation specialization involves linear development. With regard to participants in recreational sailing, Kuentzel and Heberlein (1997) confirmed that the improvement of individual self-development status is positively correlated with the level of specialization with regard to sailing participation. Oh et al. (2013) as well as other scholars have also conducted research from the perspective of predictive development, constructed a structural model based on a survey of fishing professionals in Texas, and found that as fishing specialization increases, anglers’ attachment to the program also increases. However, some scholars have found that recreation specialization is the result of a combination of internal and external factors and that its complexity does not completely comply the law of linear development. Scott and Godbey (1994) first raised doubts, and in his in-depth research on bridge players, he found that over time, some players did not achieve elite status but were rather content to continue to play the game at a basic level. In addition to Scott and Lee (2010) points, it has been claimed that accidental events and changes in participation throughout the life course can also have disturbing effects on the predictive capacity of recreation specialization indicators.

These nonlinear manifestations have also made recreation specialization measures and the scope of relevant research more diverse. Beardmore et al. (2013) used a life-centric approach to examine the specialization of anglers in a certain region in Germany, introducing specific indicators such as skills, mediums, rewards, and catch receipts to predict fishing behavior and preference. Jun et al. (2015) focused on individual performance in the context of recreation and used cognitive, behavioral, and affective variables to assess participants’ level of specialization. Bricker and Kerstetter (2000) combined with the concept of local attachment, five scales pertaining to experience level, centrality of lifestyle, degree of participation, skill level and expenditure level were constructed, and the degree of specialization of participants in whitewater recreation in the western United States was studied. To evaluate the impact of different degrees of injury among athletes on leisure patterns, Ioseba Iraurgi (2021) evaluated the composite dimensions of deep leisure and recreation specialization using the Deep Leisure Scale (SLIM) and the Recreation Specialization Index (RSI), Groups of participants without physical disabilities were found to exhibit higher levels of recreation specialization. As the research on recreation specialization matured, Scott and Shafer constructed a recreation specialization evaluation index, that is, a three-dimensional measurement model of recreation specialization featuring movement, skills, knowledge and commitment (Scott and Shafer, 2001). This model has been widely used in leisure research. Hitherto, international studies have been conducted to investigate specialization in the context of skiing and recreation. Leisure constraints have a positive impact on recreation specialization (Hwang, 2013). Won et al. (2008) survey regarding ski recreation found that the level of specialization has an important impact on consumption and destination preferences with regard to snow resorts. Based on the late start of the development of ski resorts in China, the relevant research is not yet mature.

## 3 Research hypotheses

### 3.1 Hypothesis regarding motivation as an antecedent variable

Jackson et al. (1993) introduced the psychological structure of leisure motivation into the hierarchical constraint model in the form of “equilibrium propositions.” Carroll and Alexandris (1997) explained this notion by reference to the fact that highly motivated individuals tend to perceived high levels of constraint less often and are more likely to engage in leisure activities, and he suggested a correlation between these factors rather than merely causation, i.e., a negative bivariate correlation between motivation and constraint. Chen et al. (2013) conducted a survey on the leisure activities of Taiwanese teenagers to encourage participation in leisure activities with the goal of enhancing the physical and mental health of adolescents and found a negative correlation between leisure motivation and leisure constraints. Based on the leisure constraints model, Gage and Thapa (2012) took college student volunteers in the southeastern United States as a sample and found that leisure motivation negatively affects leisure constraints, while and structural restriction is more significant. Lee and Newpaney (2015) claimed that motivation helps people develop leisure skills, which in turn helps reduce cost constraints for participants. However, Liu and Walker (2015) came to a conclusion that is inconsistent with those of the scholars mentioned above, finding that motivation and constraints are positively correlated with constraints based on their investigation of the impacts of motivation, restriction and urbanization factors on the leisure time physical activity of Chinese residents. This view reflects the results regarding leisure activity among elderly people in China (Yi, 2009), indicating that the higher the requirements for participation are, the more obstacles are encountered. Accordingly, this paper posits that the attractiveness of ice and snow leisure sports stimulates the public’s motivation to participate; however, the outdoor conditions, technical thresholds, capital requirements and other factors necessary for ice and snow sports represent restricted content. Accordingly, this paper proposes that the leisure motivation underlying ice and snow participation has a negative impact on such restriction; that is, the stronger the motivation to participate in ice and snow is, the less restricted content is observed.

*H*1: Leisure motivation has a negative impact on leisure constraints.

Edger argued that motivation, as a precursor of engagement in leisure activities, plays a central role in the process of leisure constraint negotiation and has a positive impact on alternative negotiations (Jackson et al., 1993). This finding was confirmed by Chun et al. (2022), who used Canadian and Korean hikers as control groups and found that the relationship between motivation and negotiation was positively significant. Kono et al. (2020) studied the leisure activities of Japanese and European people and drew the same conclusion. These findings demonstrate the direct relationship between the leisure motivations and adaptations of groups drawn from different cultural backgrounds. This study posits that when people engaging in ice and snow sports leisure activities have a strong motivation to participate, they propose more strategies to overcome difficulties; thus, this paper proposes the hypothesis that leisure motivation has a positive impact on leisure flexibility.

*H*2: Leisure motivation positively influences leisure flexibility.

Chou (2020) statistical analysis of partial least squares (PLS) showed that cyclists exhibit higher motivation and a higher level of recreation specialization. Randler (2023) univariate analysis found significant differences between members and nonmembers in all dimensions; in addition, the stronger the motivation was, the higher the coefficient level, indicating that leisure has a positive effect on recreational specialization. Similarly, controlled experiments have been conducted to show that motivation has a driving effect on participation in leisure. However, with regard to different motivations, highly professional anglers and general anglers exhibit differences; the former cite strong resource-related motivations (seeking trophy fish, fishing techniques, etc.), while the latter participate due to their desire for primary recreation (Chipman, 1988). Therefore, when the person who may engage in leisure activities has a strong desire to participate, his participation increases, and in this process, he becomes more proficient at using the relevant technology through repeated training and develops a deep understanding of leisure projects, thereby strengthening his level of specialization. Accordingly, this study proposes that leisure motivation can positively affect specialization in the context of ice and snow sports recreation.

*H*3: Leisure motivation positively influences recreation specialization.

### 3.2 Leisure workaround as an antecedent variable

Most leisure scholars have claimed that the use of alternative resources or strategies can reduce the negative impact of restrictions on participation when constraints are encountered (Hubbard and Mannell, 2001). In Xie and Ritchie (2019) travel research, he argued that the use of workarounds reduces the negative impact of travel constraints on travel intent only partially, as many other factors affect the strength of workarounds, such as the perceived strength of travel restrictions. Zhou et al. (2017) summarized five alternative strategies in a qualitative study of marathon participants to effectively overcome the limiting factors associated with marathon events. Qiu et al. (2018) claimed that at different stages of leisure participation, individuals employ different workaround strategies to avoid or reduce the impact of restrictions, thereby also confirming the mitigating effect of workarounds on restrictions. Therefore, this study posits that although there ice and snow sports feature a great deal of restrictive content, the threshold for participation can be effectively lowered using alternative methods.

*H*4: Leisure negotiation have a mitigating effect on leisure constraints.

### 3.3 Leisure constraints as antecedent variables

Using a cluster sampling method, Chou (2020) selected 374 skiers from Gyeonggi and Gangwon provinces and found that leisure flexibility (constraint negotiation) has a positive effect on recreation specialization. Kim (2011) research regarding Seoul’s commercial sports center group research found that recreation specialization progresses when people struggle to negotiate with leisure constraints, illustrating that effective adaptations can have positive effects on recreation specialization. These authors found that the relationship between flexibility and engagement was significant only in the European-Canadian sample but not in the Japanese sample, suggesting that the strength of the workaround also influenced leisure outcomes (Kono and Ito, 2021). Chun et al. (2022) also performed a controlled experiment to find the difference between Koreans and Canadians in the context of trekking: under the same conditions, Canadians facilitated their trekking activities using alternative strategies, while Koreans faced difficulty with regard to overcoming certain obstacles, indicating that alternative strategies, as mediators, affect constraints and participation.

*H*5: Leisure flexibility positively affects recreation specialization.

Leisure scholars have claimed that when leisure activities encounter restrictions and obstacles, leisure participation is reduced, which is not conducive to recreation specialization. Park (2017) research on the professional activities of water leisure in Busan concluded that two subfactors of leisure constraints, i.e., personal constraints and structural constraints, have negative impacts on entertainment specialization, while interpersonal constraints are not significant in this context. Liu and Lou (2016) concluded based on the behavior of camping enthusiasts that the higher the level of leisure constraints is, the stronger the negative impact on recreation specialization. Accordingly, this study proposes that in the context of ice and snow sports, this limiting factor impedes leisure specialization.

*H*6: Leisure constraints negatively affects recreation specialization.

### 3.4 The intermediary role of leisure negotiation

Qiu (2009) claimed that the relationship between motivation and restriction also differs across different stages of the development of recreational sports behavior and that individuals employ different alternative strategies to coordinate the relationship between these two factors. Xie and Ritchie (2019) introduced leisure theory to the study of travel behavior, arguing that strongly motivated groups tend to use negotiation strategies more frequently to facilitate final destination selection and that the main influence of travel motivation on travel intention is indirect, i.e., it is mediated through alternative strategies; in addition, Xie specifically explained that the more travel restrictions individuals encounter, the more likely they are to use negotiation strategies to overcome these restrictions. Hubbard and Mannell (2001) agreed that negotiation has been found to play a mediating role in the mechanism underlying the influence of motivation and participation. Recreation specialization represents a deepening of leisure participation; thus, this study posits that leisure negotiation plays a mediating role in this context, and when it operates as such an intermediary mechanism, leisure motivation and leisure constraints have positive impacts on recreation specialization. Accordingly, the following hypotheses are proposed:

*H*7: Leisure negotiation have a mediating effect on the relationship between leisure motivation and leisure specialization.

*H*8: Leisure flexibility has a mediating effect on the relationship between leisure constraints and leisure specialization.

## 4 Method

### 4.1 Participants and procedure

Taking 8 indoor and outdoor skating rinks and 7 ski resorts in Beijing as examples, this study took tourists who were directly involved in ice and snow sports as its target research group and employed a random and convenience sampling method to facilitate the distribution, completion and collection of on-site questionnaires to investigate people participating in ice and snow sports. Between January 3 and 10, 2021, a total of 1,000 questionnaires were distributed, and 914 were collected (from 474 skating rink participants and 440 ski resort guests); the questionnaire recovery rate was 91.4%. After a consistency check, 108 invalid questionnaires were eliminated, and 806 valid questionnaires were retained (including 405 from the skating rink and 401 from the ski resort), for an effective rate of 88.2%. The sample characteristics are shown in Table 1, including those of ice and snow sports participants in terms of gender, age, educational background, years of participation, annual participation frequency, and single-session duration.

**Table 1:** Statistical characteristics of the sample of ice and snow sports participants.

Sample features	Classification	Participant characteristics		Sample features	Classification	Participant characteristics	
		Sample size	Proportion			Sample size	Proportion
Gender	Male	402	49.90%	Years of participation	Less than 1 year	474	58.80%
	Female	404	50.10%		1–2 years	165	20.50%
Age	Under the age of 18	32	4.00%		3 years and above	167	20.70%
	18–24 years old	359	44.50%		Never participate	122	15.10%
	25–35 years old	305	37.80%	Annual participation frequency	1–2 times	429	53.20%
	36–45 years old	90	11.20%		3–4 times	119	14.80%
	Over 46 years old	20	2.50%		More than 5 times	136	16.90%
Educational attainment	Senior high school and below	47	5.80%		Within 30 min	70	8.70%
	Junior college	78	9.70%	Single-session duration	30–60 min	153	19.00%
	Bachelor’s degree	425	52.70%		1–2 h	207	25.70%
	Postgraduate	256	31.80%		2–3 h	106	13.20%
					3–4 h	144	17.90%
					More than 4 h	126	15.60%
Total		806	100%	Total		806	100%

### 4.2 Variable measurement

The questionnaire mainly measured the four latent variables included in the conceptual model (leisure motivation, leisure constraints, alternative strategies, and leisure specialization). All scales refer to relevant research results obtained both at home and abroad, which have been adapted to suit the characteristics of China’s sports culture and ice and snow projects, thereby making them more suitable for the specific conditions of ice and snow sports participants in China. The “leisure motivation” variable in the scale is based mainly on the leisure motivation scale developed by Beard and Ragheb (1983). The variable for “leisure constraints” refers mainly to Gery and Dong (2009) and Qiu et al. (2012). This factor was divided into three dimensions: personal constraints, interpersonal constraints, and structural constraints; The “leisure negotiation “mainly refers to Loucks-Atkinson and Mannell (2007), Kim et al. (2008) and Qiu et al. (2018), this factor was divided into five dimensions: help relationship, funding adjustment, time and intensity adjustment, enhancement skills, and environmental support; The measure of recreation specialization refers to the scale developed by Scott and Shafer (2001), which includes the three dimensions of cognition, action, and affect; each variable is measured using a classic 5-point Likert scale (1 = strongly disagree, 5 = strongly agree).

## 5 Data analysis

### 5.1 Data reliability and validity results

To ensure the validity and scientificity of the data, the questionnaire results were tested using SPSS 26.0 software, and the KMO value was 0.90. The results of each index are shown in Table 1, which indicates that the standardized factor load of all observed variables was greater than 0.6 with exception of the interpersonal constraint dimension in leisure constraints; these findings indicate that the observed variables have high explanatory value. The Cronbach’s *α* coefficients for the four leisure variables were 0.888 for leisure motivation, 0.869 for leisure constraint, 0.88 for leisure variant, and 0.941 for recreational specialization. The internal consistency of each dimension is thus high, indicating that the data are relatively reliable. The results of confirmatory factor analysis showed that with the exception of the AVE value of the leisure constraint dimension, which was 0.409, the mean variance extraction (AVE) values of the remaining components ranged between 0.481–0.641. The combined reliability (CR) values ranged between 0.771 and 0.842, and the reference standard higher than 0.5 indicated that the convergence validity of the data results was good. The structural validity test of the model was performed using AMOS 26 software, and the overall fit degree test is shown in the table. The chi-square degree of freedom ratio (X^2^/df) was 2.486, with an approximate root mean square error index (RMSEA) of 0.043; comparative fit index (CFI), canonical fit index (NFI), and Tucker–Lewis index were also calculated. The results of several indicators of index (TLI) were close to 0.9 or greater than 0.9, thus meeting the basic conditions for adaptation.

### 5.2 Second-order factor pathway test results

**Figure 2:**
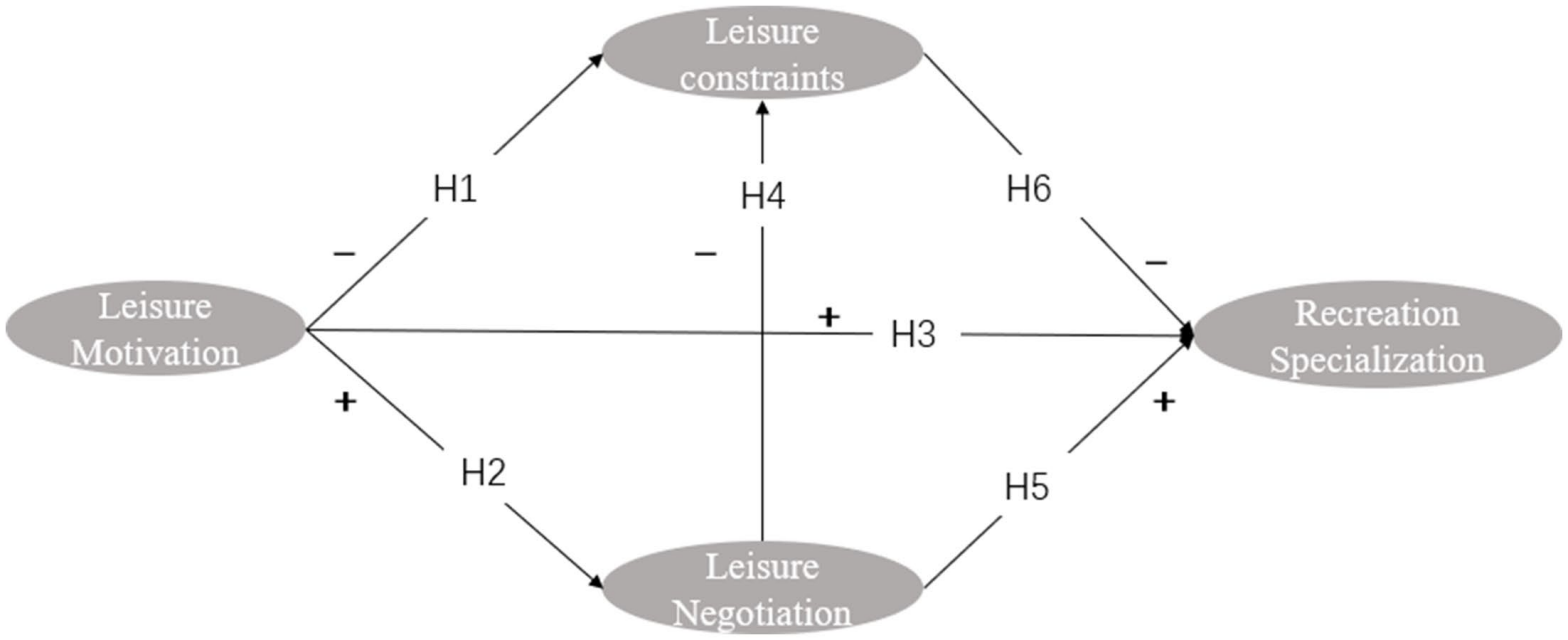
Conceptual model of ice and snow leisure behavior.

The standardized path coefficients for the structural equation model are shown in Table 2 and Figure 2. Leisure motivation has a negative effect on leisure constraints, and the standardization coefficient is −0.0.072 (*p* = 0.017). H1: The findings show that the stronger people’s motivation to participate in leisure activities is, the fewer constraints they face. Leisure motivation has a positive effect on leisure negotiation, and the results are significant. The standardization coefficient is high at 0.626 (*p*<0.001), indicating that driven by high motivation, people engaging in leisure activities are more willing to adopt effective alternative strategies to overcome difficulties, thus supporting H2. However, leisure motivation has no direct effect on recreation specialization, and the normalization coefficient is 0.058 (*p* = 0.421). This result is not significant, thus rejecting Hypothesis H3. Leisure flexibility has a significant negative impact on leisure constraints, and the standardization coefficient is −0.357 (*p* < 0.001), thus supporting H4. Leisure negotiation also has a positive effect on recreation specialization, and the standardization coefficient is 0.439 (*p* < 0.001), indicating that effective workarounds can promote the development of leisure professionals in the direction of specialized leisure, thereby supporting H5. Leisure constraints have a negative impact on recreation specialization, and the standardization coefficient is −0.111 (*p* = 0.02), illustrating that it is difficult for people engaging in leisure activities to achieve recreation specialization when subject to various constraints. Hypothesis H6 is thus supported.

**Table 2:** Test of data aggregation validity and reliability.

Variable	Variable dimensions	Factor loading	Cronbach’s *α*	AVE	CR
Leisure motivation	Intellect	0.863	0.888	0.5509	0.828
	Society	0.624			
	Skill	0.773			
	Stimulus avoidance	0.687			
Leisure constraints	Personal constraints	0.475	0.869	0.409	0.772
	Interpersonal constraints	0.728			
	Time constraints	0.587			
	Economic constraints	0.735			
	Environmental constraints	0.636			
Casual adaptation	Helpul relationship	0.755	0.88	0.4816	0.787
	Funding adjustments	0.703			
	Time and intensity adjustment	0.656			
	Enhance skills	0.657			
Recreation specialization	Cognition	0.789	0.941	0.641	0.843
	Action	0.83			
	Emotion	0.782			

X^2^/df = 2.486; RMSEA = 0.043; RFI = 0.864; IFI = 0.919; CFI = 0.918; NFI = 0.871; TLI = 0.914.

**Figure 3:**
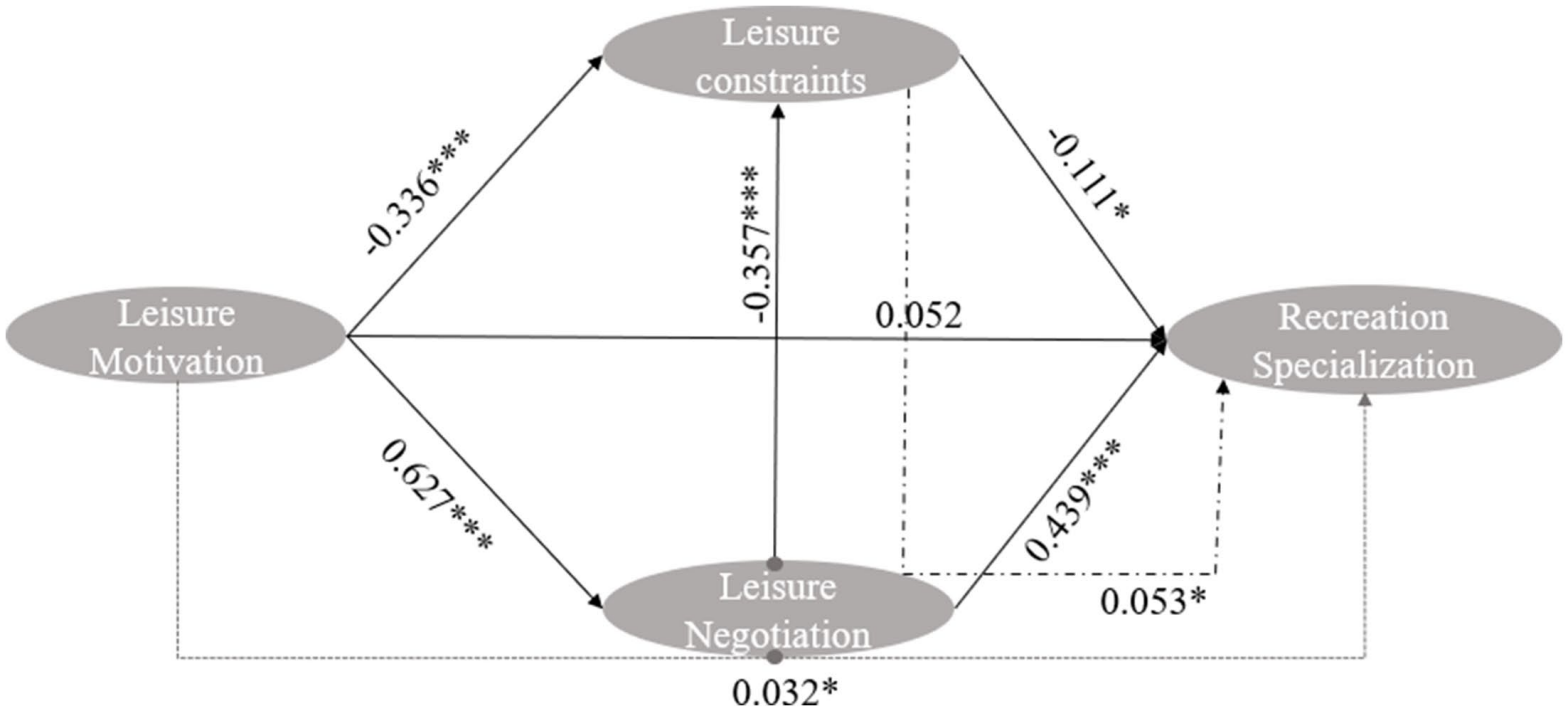
Results regarding the ice and snow leisure behavior path. ***p < 0.001; *p < 0.5.

According to the analysis conducted using AMOS 26.0, which featured leisure constraints and leisure negotiation as the mediating variables in the model, as shown in Table 3 and Figure 2, and the analysis of the standardized mediation effect, leisure motivation has a positive impact on leisure specialization at the 95% level. The CI values range from 0.21–0.398 (not including 0), and the direct path shows that leisure motivation has no direct effect on recreation specialization, indicating that leisure negotiation plays a complete mediating role in this process. Leisure constraints have a positive effect on recreation specialization through leisure negotiation, and 95% of the CI values range from 0.055 to 0.165 (not including 0); the results are thus significant. According to the direct path, leisure constraints have no positive impact on recreation specialization, indicating that leisure negotiation also has a complete mediating effect on this path relationship and that when people engaging in leisure activities encounter various constraints, they can address their difficulties and increase the possibility of deep participation through adaptation (Figure 3).

**Table 3:** Direct path inspection results.

Hypothesis	Path	Standardization coefficient	Nonnormalized coefficients	S. E.	C. R.	*p*	Outcome
H1	Casual motivation → casual flexibility	0.627	0.602	0.056	10.792	***	Supported
H2	Leisure motivation → leisure constraints	−0.336	−0.215	0.049	−4.365	***	Supported
H3	Leisure motivation→ recreation specialization	0.052	0.058	0.072	0.805	0.421	Not supported
H4	Leisure flexibility → leisure constraints	−0.357	−0.237	0.053	4.473	***	Supported
H5	Leisure negotiation→ recreation specialization	0.439	0.505	0.084	6.036	***	Supported
H6	Leisure constraints → recreation specialization	−0.111	−0.192	0.083	−2.325	0.02*	Supported

*** *p* < 0.001; *p < 0.5.

In statistical terms, a *p*-value less than 0.01 indicates that the result is statistically highly significant, while a *p*-value less than 0.05 indicates that the result is statistically significant.

### 5.3 Advanced analysis of the influence of alternative strategies on first-order factors associated with recreation specialization

The results of the structural equation model show that the leisure negotiation has a significant positive impact on recreation specialization. To explore the specific impact of leisure negotiation on the dimension of recreation specialization in further detail, this study divided the respondents into three groups based on their length of participation and explored the specific effects of alternative strategies on the first-order factors associated with recreation specialization, which is shown in Table 4.

**Table 4:** Indirect path inspection results.

Path	SE	Effect size	Bias-corrected 95% CI			Percentile 95% CI		
			Lower	Upper	*p*	Lower	Upper	*p*
Leisure motivation→ leisure negotiation→ leisure specialization	0.053	0.286	0.21	0.398	0.002*	0.202	0.38	0.004*
Leisure constraints→ leisure negotiation→ leisure specialization	0.032	0.096	0.055	0.165	0.002*	0.051	0.157	0.004*

***p < 0.001; *p < 0.5.

In statistical terms, a *p*-value less than 0.01 indicates that the result is statistically highly significant, while a *p*-value less than 0.05 indicates that the result is statistically significant.

Overall, leisure flexibility has significant positive impacts on cognition, action and emotion in the three groups. Compared with individuals who have participated in ice and snow sports for 1–2 years, the implementation of alternative strategies by beginners in leisure-related activities (i.e., people with less than 1 year of experience) and people with more than 3 years of experience has a more significant impact on the path of recreation specialization. The specific conceptual path indicates that the alternative strategy effectively improves the “cognition” of recreation specialization, and the coefficients of participants with less than 1 year or more than 3 years of experience were higher than 0.7. The specific effectiveness coefficient with respect to “action” was the highest among participants with less than 1 year of experience, followed by participants with more than 3 years of experience, indicating that strategy implementation can effectively promote the actual participation of people in leisure activities. Similarly, the “emotional” conceptions of participants with less than 1 year of experience is also affected by the workaround, and this influence is greater than that exhibited by participants with more experience (Table 5).

**Table 5:** The impact coefficients of different years of participation in leisure activities and adaptation strategies on recreation specialization.

Path	Years of participation in snow sports		
	Less than 1 year (*N* = 474)	1–2 years (*N* = 165)	More than 3 years (*N* = 167)
Leisure flexibility → cognition	0.731***	0.584***	0.746***
Leisure negotiation → actions	0.813***	0.507***	0.731***
Casual flexibility → emotion	0.783***	0.563***	0.727***

***p < 0.001; *p < 0.5.

In statistical terms, a *p*-value less than 0.01 indicates that the result is statistically highly significant, while a *p*-value less than 0.05 indicates that the result is statistically significant.

## 6 Discussion

First, “Leisure motivation” is the internal driving force underlying ice and snow sports participation. Motivation is negatively correlated with “leisure constraint,” and high participation motivation can effectively alleviate internal and external constraints in the leisure process. One positive impact path involves “leisure negotiation,” which is driven by motivation, and people who engage in ice and snow activities for the sake of leisure employ a variety of strategies to mitigate or overcome constraints. However, motivation has no direct impact on “recreational specialization,” indicating that motivation affects short-term participation behavior, while long-term professional behavior in specialized fields relies solely on motivation as the only relevant factor.

Second, “leisure negotiation” is an important measure to promote the long-term development of mass ice and snow participation. When included as a leading variable, leisure flexibility is negatively correlated with constraints. The implementation of a leisure negotiation is not limited to fixed content but can have marginal effects that mitigate other constraints. Leisure negotiation has a positive effect on recreation specialization. When leisure negotiation is included as an intermediary variable, it has a complete mediating effect on the relationships between leisure motivation and recreation specialization and between leisure constraints and recreation specialization, indicating that the implementation of alternative strategies has the function of connecting the top and bottom.

Third, the restrictive content associated with ice and snow sports hinders the sustainable development of the ice and snow industry. A significant negative relationship is observed between leisure constraints and recreation specialization, and the existence of these factors greatly limits specialization and deep participation in the context of ice and snow, which is the content to which relevant managers must pay a great deal of attention.

Fourth, the influence coefficients of alternative strategies on the recreation specialization of people who engage in leisure activities and exhibit different levels of participation differ. As people’s durations of participation change, the change trend of “high-low-high” impact degree emerges.

To enhance ice and snow sports’ popularity and professionalism, venue operators should adopt targeted strategies based on above findings, focusing on participants’ motivation and adaptive tactics. Multi-perspective analysis of leisure participants’ motivation is crucial to boosting public engagement and in-depth participation, with specific approaches as follows: Firstly, emphasize individual internal motivation. Data show “intellectual motivation” is key, as participants engage for learning, relaxation, interest, and achievement. Secondly, support external motivation. Driven by social needs, participants use these sports for interaction. Suppliers can build online-offline platforms, hold regular friendly competitions, encourage experience sharing, and strengthen local communication. Also, establish hierarchical marketing for varying involvement levels: venues can interconnect, record participation frequency and preferences via big data, and implement precision marketing.

## Data Availability

The raw data supporting the conclusions of this article will be made available by the authors, without undue reservation.
